# Carotenoids

**DOI:** 10.1016/j.advnut.2024.100304

**Published:** 2024-09-18

**Authors:** Abdulkerim Eroglu, Genan Wang, Nathan Crook, Torsten Bohn

**Affiliations:** 1 Department of Molecular and Structural Biochemistry, College of Agriculture and Life Sciences, North Carolina State University, Raleigh, NC, United States; 2 Plants for Human Health Institute, North Carolina Research Campus, North Carolina State University, Kannapolis, NC, United States; 3 Department of Chemical and Biomolecular Engineering, College of Engineering, North Carolina State University, Raleigh, NC, United States; 4 Nutrition and Health Research Group, Department of Precision Health, Luxembourg Institute of Health, Thomas Edison, Strassen, Luxembourg

## Nutrient

This article is an updated version of a previous publication about carotenoids [1]. Carotenoids are naturally occurring colorful pigments belonging to a subset of terpenoids, mostly tetraterpenoids. Following consumption of a carotenoid-rich diet, that is, a diet rich in colored fruits and vegetables, they are first released from the food matrix and incorporated into mixed micelles in the intestinal lumen, followed by their uptake by intestinal mucosal cells via passive diffusion or lipid transporters, depending on the dose. Next, they are incorporated into chylomicrons and released into the lymph [2]. Carotenoids have important antioxidant, immunologic, and metabolic functions owing to their interactions with inflammatory transcription factors and oxidative stress pathways [3]. They are likely implicated in mitigating inflammation in diseases characterized by low-grade inflammation, such as obesity [4]. Although biological activities and health benefits of some carotenoids are attributed to their conversion to vitamin A, others, such as lutein and zeaxanthin, can enhance cognitive and visual function, as shown in a recent randomized controlled trial [5]. Although absorbed carotenoids display health benefits, as described earlier and detailed in the following sections, carotenoids entering the large intestine may also impact host metabolism via interacting with the microbial communities in the gut, as extensively summarized by our groups [6]. The most frequently consumed carotenoids in the regular diet include lycopene, β-carotene, α-carotene, lutein, β-cryptoxanthin, and zeaxanthin, with a reported total daily intake of ∼12 mg [7]; however, phytoene and phytofluene, as well as neoxanthin and violaxanthin and further the algae carotenoids canthaxanthin, fucoxanthin, and astaxanthin are also, though to a lesser degree, consumed. Lycopene and β-carotene constitute the most abundant carotenoids in human plasma, with a sum of their concentrations of typically 1–2 μM [7].

## Deficiencies

No specific deficiency is associated solely with low carotenoid intake; hence, carotenoids are not considered essential and are not termed nutrients but rather bioactive compounds. However, several carotenoids, including α-carotene, β-carotene, and β-cryptoxanthin, are provitamin A carotenoids that can replace preformed vitamin A—in this sense, they may be essential for vegetarians. In addition, there is some evidence for the contribution of lutein and zeaxanthin to visual acuity and the prevention of age-related macular degeneration [8]. Thus, their semiessential character has been discussed [9] but is lacking consensus.

## Dietary Recommendations

There exist no dietary reference intakes by USDA or population reference intakes by European Food Safety Authority (EFSA). A recommendation for β-carotene exists in the absence of preformed vitamin A intake; that is, 3.9/4.5 mg/d for females/males would be equivalent to the 0.65/0.75 mg vitamin A (retinol equivalents) intake recommendation given a conversion factor of 1:6 (of β-carotene to retinol) [10]. Some countries, such as China and Korea, promote the intake of lutein at 10 mg/d. No tolerable upper intake level has been defined. However, EFSA now recommends smokers to avoid supplemental β-carotene [10]. Acceptable daily intake levels for lutein (from food additives/supplements) and lycopene (from all sources) by EFSA of 1 mg/kg and 0.5 mg/kg body weight and day, respectively, have been reported.

## Clinical Uses

Clinical uses are limited. However, ophthalmologists frequently prescribe lutein and zeaxanthin to improve visual acuity, especially during advanced age, for example, 10 and 2 mg/d, respectively [11]. Furthermore, mixed carotenoid supplementation has been advocated for photoprotective reasons and skin protection [12].

## Toxicity

In general, no toxicity of high intakes of carotenoids has been reported, and no upper intake levels have been defined for any of the carotenoids. High canthaxanthin intake can increase risk of retinopathy [13]. A high intake of β-carotene has been reported to cause carotenodermia, a reversible skin coloration [14]. Moreover, β-carotene increased lung cancer rate in smokers when supplemented at high concentrations (>20 mg/d), especially when leading to plasma concentrations above 3.9 μM as reviewed by EFSA [10], for reasons not completely understood. In addition, based on appropriate animal models (gerbils), it may be argued that β-carotene could contribute to high liver vitamin A stores when consumed concomitantly with high amounts of vitamin A, potentially contributing to toxicity [10].

## Recent Research

Recent research included the prevention of age-related macular degeneration [8] and the general investigation of carotenoids concerning cardiometabolic health [15]. Owing to their low and variable absorption, bioavailability studies have investigated the interaction of carotenoids with divalent minerals [16] or proteins [17]. New sources of carotenoids, such as from algae and, more recently, from insects, [18] are also prominent topics. Because of the general health recognition of the gut microbiome (GM), the interaction of carotenoids and GM has also received recent attention [6]—a number of new trials on GM-cognition are underway [19]. Regarding the GM, multiple studies have investigated the impact of carotenoids on the microbial community structure. It was found that astaxanthin could prevent alcoholic fatty liver disease by modulating GM composition in mice [20]. The prebiotic effect of carotenoids was noted in an intervention trial in adults with obesity, as the relative abundance of *Bifidobacterium adolescentis* and *Bifidobacterium longum* increased upon lycopene supplementation [21]. Karlsson et al. [22] discovered that the GM could reduce atherosclerosis by elevating β-carotene concentrations in the serum via higher activity of phytoene dehydrogenase enzyme, a rate-limiting step for carotenoid biosynthesis, found in the microbiota of healthy individuals compared with that of atherosclerotic patients [22]. Many carotenoid studies also showed a GM-dependent impact on human gene expression, such as scavenger receptor class B type I [23]. In an in vitro fermentation model, the supplementation of β-carotene to GM benefited vitamin A formation and induced the microflora to produce more short-chain fatty acids [24]. Grizotte-Lake et al. [25] found that GM regulated the expression of retinol dehydrogenase 7 (*Rdh7*), indicating that microbial communities can impact retinoid metabolism. Furthermore, Bonakdar et al. [26] showed that GM possessed retinoid metabolic activity as microbial communities in the gut restored retinoic acid levels in the gut of vancomycin-treated mice. These exciting studies indicate that there is still an unrevealed potential of dietary carotenoids, motivating deeper mechanistic studies in carotenoid and microbiome research.

## Author contributions

The authors’ responsibilities were as follows: AE prepared the outline for the overall manuscript, and all authors were involved in the conception and writing of the manuscript and have read and approved the final content.

## Funding

No funding was received for this manuscript. AE and NC received funds from the USDANIFAAFRI Foundational and Applied Science Program (A1343—Food and Human Health), Grant Award Number: 2022-67018-37188.

## Conflicts of interest

The authors report no conflicts of interest.
